# Venous and Arterial Responses to Partial Gravity

**DOI:** 10.3389/fphys.2020.00863

**Published:** 2020-07-28

**Authors:** Stuart M. C. Lee, David S. Martin, Christopher A. Miller, Jessica M. Scott, Steven S. Laurie, Brandon R. Macias, Nathaniel D. Mercaldo, Lori Ploutz-Snyder, Michael B. Stenger

**Affiliations:** ^1^KBR, Houston, TX, United States; ^2^Memorial Sloan Kettering Cancer Center, New York, NY, United States; ^3^School of Kinesiology, University of Michigan, Ann Arbor, MI, United States; ^4^Lyndon B. Johnson Space Center, National Aeronautics and Space Administration, Houston, TX, United States

**Keywords:** internal jugular vein, parabolic flight, spaceflight-associated neuro-ocular syndrome, venous thrombosis, artificial gravity, gravity levels

## Abstract

**Introduction**: Chronic exposure to the weightlessness-induced cephalad fluid shift is hypothesized to be a primary contributor to the development of spaceflight-associated neuro-ocular syndrome (SANS) and may be associated with an increased risk of venous thrombosis in the jugular vein. This study characterized the relationship between gravitational level (G_z_-level) and acute vascular changes.

**Methods**: Internal jugular vein (IJV) cross-sectional area, inferior vena cava (IVC) diameter, and common carotid artery (CCA) flow were measured using ultrasound in nine subjects (5F, 4M) while seated when exposed to 1.00-G_z_, 0.75-G_z_, 0.50-G_z_, and 0.25-G_z_ during parabolic flight and while supine before flight (0-G analog). Additionally, IJV flow patterns were characterized.

**Results**: IJV cross-sectional area progressively increased from 12 (95% CI: 9–16) mm^2^ during 1.00-G_z_ seated to 24 (13–35), 34 (21–46), 68 (40–97), and 103 (75–131) mm^2^ during 0.75-G_z_, 0.50-G_z_, and 0.25-G_z_ seated and 1.00-G_z_ supine, respectively. Also, IJV flow pattern shifted from the continuous forward flow observed during 1.00-G_z_ and 0.75-G_z_ seated to pulsatile flow during 0.50-G_z_ seated, 0.25-G_z_ seated, and 1.00-G_z_ supine. In contrast, we were unable to detect differences in IVC diameter measured during 1.00-G seated and any level of partial gravity or during 1.00-G_z_ supine. CCA blood flow during 1.00-G seated was significantly less than 0.75-G_z_ and 1.00-G_z_ supine but differences were not detected at partial gravity levels 0.50-G_z_ and 0.25-G_z_.

**Conclusions**: Acute exposure to decreasing G_z_-levels is associated with an expansion of the IJV and flow patterns that become similar to those observed in supine subjects and in astronauts during spaceflight. These data suggest that G_z_-levels greater than 0.50-G_z_ may be required to reduce the weightlessness-induced headward fluid shift that may contribute to the risks of SANS and venous thrombosis during spaceflight.

## Introduction

When a person stands upright on Earth, gravity pulls body fluids (e.g., blood, lymph, and cerebrospinal fluid) toward their feet, resulting in approximately 70% of body fluids residing below heart level (Rowell, 1993). During spaceflight, the absence of a head-to-foot hydrostatic pressure gradient causes these fluids to redistribute, resulting in a fluid shift toward the head (Thornton et al., 1987). These fluid shifts toward the head during weightlessness initially cause rapid alterations in the cardiovascular system, particularly in the venous circulation (Martin et al., 2016) as well as the cerebrospinal fluid (Lawley et al., 2017). Chronic exposure to weightlessness without countermeasures results in cardiovascular deconditioning (Hargens and Watenpaugh, 1996) and regional adaptations in the blood vessels (Zhang, 2001).

Long-duration stays in weightlessness also have resulted in changes in the function and structure of the eye in some astronauts that has been described as spaceflight-associated neuro-ocular syndrome (SANS; Lee et al., 2016; Macias et al., 2020). The leading hypothesis is that ocular changes result from chronic exposure to the weightlessness-induced fluid shift (Stenger et al., 2017). A consequence of the headward fluid shift appears to be congestion of the veins that drain the head (Arbeille et al., 2015; Marshall-Goebel et al., 2019). This, in turn, may impair cerebrospinal and lymphatic fluid drainage from the skull (Macintyre, 2013), which may underlie some of the changes in the eye. Previous work demonstrated that jugular vein cross-sectional area (Arbeille et al., 2015) and pressure (Marshall-Goebel et al., 2019) are increased in weightlessness (0-G) relative to the upright posture in normal gravity (1.00-G_z_). Further, we have recently documented IJV flow pattern changes that might contribute to an increased risk of venous thrombosis (Marshall-Goebel et al., 2019).

Reversing the headward fluid shift has been proposed as a method to relieve venous congestion associated with weightlessness to mitigate the risk of SANS and venous thrombosis. One approach for achieving this effect is to create artificial gravity by centrifugation (Clément et al., 2015). However, how much gravity is required to sufficiently shift fluids footward is unresolved. Parabolic flight provides a unique opportunity to evaluate the acute changes associated with varying levels of gravity (G_z_-levels) and to describe how vascular parameters change at levels less than 1.00-G_z_. Characterizing these physiological changes in response to varying G_z_-levels is an important step in determining what G_z_-level may be required to reverse weightlessness-induced fluid shifts to serve as a viable countermeasure during long-duration spaceflight. Further, this information might provide the basis for a prediction of whether G_z_-levels experienced on the Moon and Mars will be sufficient to prevent SANS development during exploration missions.

## Materials and Methods

### Overall Protocol

This study characterized a set of vascular parameters at different G_z_-levels and hydrostatic gradients, including preflight supine (1.00-G_z_ supine), 0.25-, 0.50-, and 0.75-G_z_ while seated during parabolic flight, and 1.00-G_z_ seated during level flight between parabolas. Weightless parabolas (0-G) were not included in this parabolic flight campaign. Vascular measurements included internal jugular vein (IJV) cross-sectional area, flow characterization, and pressure; common carotid artery (CCA) cross-sectional area and flow; and inferior vena cava (IVC) diameter using ultrasound. Beat-to-beat finger blood pressure and heart rate from a three-lead ECG configuration (Finapres Medical Systems, Amsterdam-Zuidoost, Netherlands) also were acquired. Study protocols were reviewed and approved by the French National Comité de Protection des Personnes and the NASA Johnson Space Center Institutional Review Board. This study was one of 11 experiments included in the parabolic flight campaign managed by Novespace, Inc. (Bordeaux-Mérignac, France) as part of the first International Space Life Sciences Working Group Campaign in June 2018.

**Figure 1:**
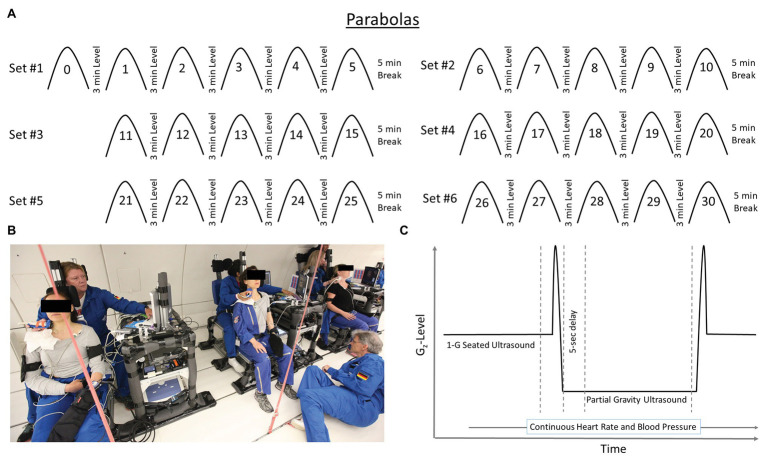
Each flight day consistent of 31 parabolas (**Panel A**) producing partial gravity levels of 0.25-, 050-, and 0.75-G_z_. Data were not analyzed for the first parabola (parabola 0) as it was considered a practice parabola for operators and subjects. Specially-designed chairs (**Panel B**) were constructed to accommodate the sonographer positioned directly behind the test subjects during the flights. Such an alignment was necessary to acquire high-quality ultrasound images. Subjects and operators wore lap belts to prevent excessive upward movement in partial gravity, and subjects rested against a back pad that extended above the level of their head. Additionally, a pad on the left side of the head allowed the subjects to relax their neck muscles. Hardware racks were positioned next to each subject such that the ultrasound machine was readily available to the sonographer, and other hardware for the measurement and recording of heart rate and blood pressure located such that the study’s engineer could monitor them. Within a parabola (**Panel C**), data were collected and analyzed during level flight before the hypergravity phase of the parabola and after 5 s of partial gravity. Depiction of time on *x*-axis and G_z_-level on the *y*-axis are not to scale.

Each flight included 31 parabolas (Figure 1), with the first parabola serving as a “practice” parabola to verify equipment operation. Then six sets of five parabolas were performed, each set at one of three different G_z_-levels (0.25-G, 0.50-G, and 0.75-G_z_; Table 1). The order of the G_z_-levels was not randomly assigned within or across days of flight but differed across days. Each parabola started with a pull-up and ended with a “pull-out” maneuver (hypergravity phase to enter and exit the parabolic flight profile) at 1.8-G, both lasting about 20 s. Accelerometer data measured in the cockpit of the plane by Novespace and collected by our group independently in the experiment area (APDM, Inc.; Portland, OR) were used to identify the respective partial gravity epochs within which data were analyzed. The air pressure in the cabin was maintained at approximately 600 mmHg (800 mbars) during the parabolas, which corresponded to an altitude of about 2,000 m. The temperature was controlled to be between 20 and 25°C.

**Table 1:** The order of G_z_-levels within and across days was not randomized, but the order of G_z_-levels varied across days.

	Parabolas	G_z_-Level	Ultrasound measures
Day 1	0–5	0.25	IJV pressure and area
	6–10	0.50	IJV pressure and area
	11–15	0.75	IJV pressure and area
	16–20	0.25	IJV flow, CCA flow and diameter, IVC diameter
	21–15	0.50	IJV flow, CCA flow and diameter, IVC diameter
	26–30	0.75	IJV flow, CCA flow and diameter, IVC diameter
Day 2	0–5	0.50	IJV pressure and area
	6–10	0.75	IJV pressure and area
	11–15	0.25	IJV pressure and area
	16–20	0.50	IJV flow, CCA flow and diameter, IVC diameter
	21–15	0.75	IJV flow, CCA flow and diameter, IVC diameter
	26–30	0.25	IJV flow, CCA flow and diameter, IVC diameter
Day 3	0–5	0.75	IJV pressure and area
	6–10	0.25	IJV pressure and area
	11–15	0.50	IJV pressure and area
	16–20	0.75	IJV flow, CCA flow and diameter, IVC diameter
	21–15	0.25	IJV flow, CCA flow and diameter, IVC diameter
	26–30	0.50	IJV flow, CCA flow and diameter, IVC diameter

### Subjects

Potential test subjects were identified by Novespace based upon selection criteria provided by the investigators. Subjects had to be between 25 and 55 years old to be similar in age to the astronaut corps (Harm et al., 2001), but there was no preferential recruitment by sex. Subjects had to be between 157 and 183 cm tall (61 and 72 in) to be secured safely in the seat. Preference was given for subjects with previous parabolic flight experience to increase the likelihood that the variable G-levels would be tolerated. Subjects were required to be French citizens to qualify for medical and life insurance in the event of an emergency but also needed to have sufficient command of English, so that the test operators could communicate with them.

In the week before the flights, subjects were screened by one of the investigators (DSM) to ensure adequate visualization of the relevant anatomy with ultrasound within the time constraints of each parabolic maneuver. Nine subjects (5F, 4M) were identified, with three subjects participating in testing on each of 3 flight days. Subjects were 39 ± 6 years old (mean ± SD; range: 34–50 years), 171 ± 11 cm tall (157–187 cm), and weighed 65 ± 10 kg (50–85 kg). All subjects were received verbal instruction and written documentation regarding study protocols and encouraged to ask questions before providing written informed consent. Written informed consent also was obtained from all participants for the publication of any potentially identifiable images.

All but one subject was administered an antiemetic (subcutaneous scopolamine, 0.25 mg/ml saline) under the supervision of the flight medical doctor before boarding the airplane. Dosages administered ranged from 0.2 ml (0.05 mg) to 0.7 ml (0.175 mg), with the majority of the subjects receiving 0.5–0.7 ml (0.125–0.175 mg). Although oral scopolamine is associated with orthostatic hypotension in some individuals (Nuotto, 1983), no symptoms were reported during this study. Further, subjects did not report any motion sickness symptoms during the flights that would have precluded participation in the experiment.

### Ultrasound Measures

Within 3 h before the flight, baseline measurements were acquired while the subjects were supine. A sonographer acquired ultrasound images (Vivid q, GE Healthcare, Chicago, IL) of the right IJV for off-line analysis of cross-sectional area, Doppler flow characterization (Marshall-Goebel et al., 2019), and estimates of IJV pressure (Martin et al., 2015, 2016); right CCA for the calculation of cross-sectional area and flow; and the IVC for the measurement of diameter. IJV images were acquired just proximal to the confluence of either the facial or superior thyroid veins, IVC images were acquired 1–2 cm from the right atrium, and CCA images were acquired ~2 cm below the carotid bulb. Venous measurements were obtained at the end of a tidal expiration. The sonographer ensured that the vessel walls could be clearly visualized and marked the skin at the probe locations where these images were obtained during the preflight baseline, so that images were acquired from the same location during parabolic flight. IJV and CCA imaging and Doppler were acquired with 12–5 MHz linear array probe (12L-RS, GE Healthcare, Chicago, IL), and IVC imaging was acquired with a 4 MHz phased array probe (M4S-RS, GE Healthcare, Chicago, IL). IJV pressure was acquired with the same 12–5 MHz linear array probe attached to a VeinPress (Meridian GMBH, Bern, Switzerland) using methods previously described (Martin et al., 2015, 2016).

During the flight, subjects were seated upright to experience the stressors associated with different G_z_-levels, and the sonographers were seated directly behind them, reaching around the subject to acquire IJV, IVC, and carotid images. Both the sonographer and the subject wore a lap belt to prevent excessive upward movements when in partial gravity. Subjects rested against a pad that extended above the level of their head, and the head was supported on the left side with an additional pad that allowed the subjects to relax their neck muscles. The same sequence of ultrasound images was acquired during each of the partial gravity conditions and during level flight, with the sonographer focusing on the acquisition of 1 parameter in each parabola. Sonographers acquired 1.00-G seated ultrasound images during level flight in the minute before the 1.8-G_z_ pull-up and acquired the partial gravity images from 5 s after the start of the partial gravity period until the end of the parabola. Heart rate and blood pressure were recorded continuously.

### Data Reduction

Average values for heart rate and blood pressure were calculated for periods during level flight from 40 to 10 s prior to the hypergravity phase (1.80-G_z_ pull-up) and for periods of partial gravity from 5 s after achieving partial gravity until the pull-out. Mean arterial pressure was calculated as the average of the whole blood pressure waveform. Three separate ultrasound images were acquired for each target in each condition and stored for offline analysis. Two sonographers independently analyzed each image in a blinded fashion, and their results (cross-sectional area, diameter, and velocity time integral) were compared. When the inter-observer difference exceeded 10% for an IJV cross-sectional area measurement or a carotid measurement or if it exceeded 20% for an IVC diameter measurement, a third sonographer analyzed the image; the average of the two closest values were used for statistical analyses.

We characterized the venous blood flow within the IJV using a 1–4 grading system that incorporated direction and pattern of the Doppler signal (Marshall-Goebel et al., 2019). Continuous forward IJV flow (head to heart direction) was scored as grade 1, pulsatile forward flow was scored as grade 2, stagnant flow was scored as grade 3, and reverse flow (toward the head) was scored as grade 4. Two trained sonographers independently scored the IJV waveforms for which there was only one discrepancy between the two raters (*n* = 45; 0.75-G_z_ rater 1 = 2 and rater 2 = 1). The Cohen’s kappa associated these ratings was 0.95 (95% CI 0.84–1.00). Due to the near perfect agreement, the discrepant value from rater 1 (senior level sonographer), along with all other identical ratings, was used for this analysis.

Data were not available for all conditions for some subjects due to technical or logistical difficulties. Beat-to-beat finger blood pressure data were not available for two subjects on day 1 and one subject on day 2. IJV cross-sectional area for one subject at all G_z_-levels and IJV pressure for all subjects while seated at 0.75‐ and 1.00-G_z_ were not of adequate quality for analysis. IJV pressure measurements are particularly difficult in the seated posture in 1.00-G_z_, given that the IJV is largely collapsed in this condition. A subset of the IJV pressure data was determined to be acceptable by one of the investigators (DSM) for five subjects during 1.00-G_z_ supine and while seated at 0.25‐ and 0.50-G_z_. No acceleration data were recorded by the experiment’s accelerometer on day 2, so accelerometer data from Novespace were analyzed for that day only.

### Statistical Approach

Descriptive and graphical summaries [mean ± SD, range (minimum, maximum), and box/line plots] were computed to summarize parabolic flight by G_z_-level. Separate linear regression models were constructed to quantify the association between each outcome (heart rate, mean arterial pressure, IJV cross-sectional area, IVC diameter, CCA flow, and cross-sectional area) and G-level (1.00-G_z_ seated, 0.75-G_z_, 0.50-G_z_, 0.25-G_z_, and 1.00-G_z_ supine). G_z_-level was modeled as a series of indicator variables, where 1.00-G_z_ (seated) was defined as the referent value. Model parameters were estimated using generalized estimating equations using an independence correlation structure (GEE-Ind) to account for repeated measurements within subject. Linear combinations of parameters were computed to estimate both main effects (e.g., expected IJV cross-sectional area at 1.00-G_z_ seated) and differences (e.g., difference in the expected IJV cross-sectional area at 0.75-G_z_ vs. 1.00-G_z_ seated) along with 95% confidence intervals and *p*-values (*via* Wald tests for difference comparisons only). A logistic regression model was constructed to quantify the relationship between IJV flow ratings (1 = pulsatile forward, 0 = continuous forward) and G-level. Odds ratios (OR), 95% confidence intervals, and *p*-values were computed using GEE-Ind to summarize the comparison when comparing each partial gravity level to 1.00-G_z_ seated. IJV pressures were compared among subjects with both 0.50-G_z_ seated and 1.00-G_z_ supine conditions using a paired *t*-test. All analyses were performed using R 3.6.2 (R Core Team, 2019).

## Results

### Parabolic Flight

**Figure 2:**
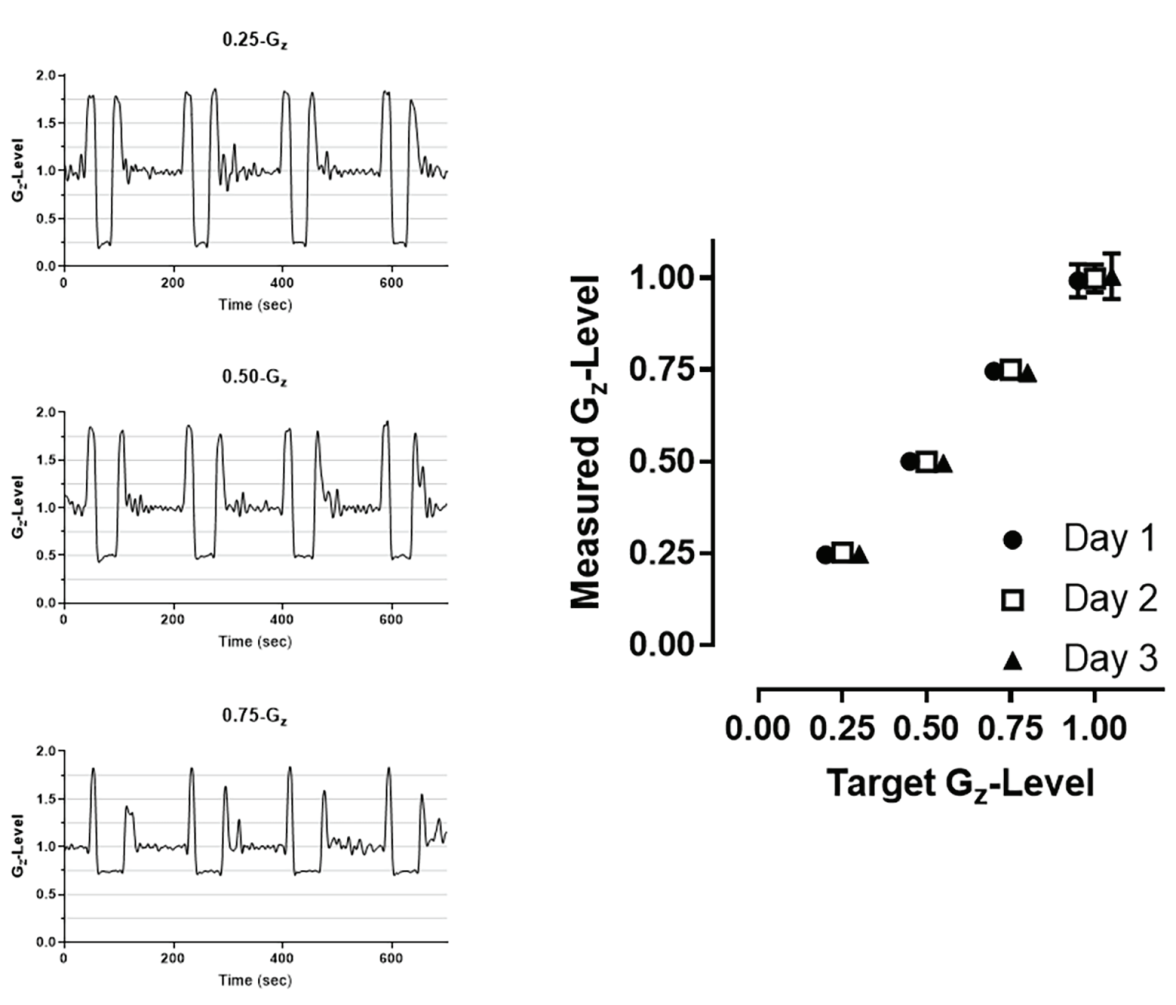
Examples of raw accelerometer data collected at each of the three G-levels (**left panels**) and mean (±SD) G-level attained across all parabolas within a given day (**right panel**). 1.00-G corresponds to level flight periods between parabolas. Error bars displaying SD may not be visible due to the low degree of variability across parabolas. Data are offset to demonstrate consistency across the 3 days (Circle: day 1; Square: day 2; Triangle: day 3).

The duration of the reduced gravity periods depended on the gravity level, with mean parabola durations of 22.1 ± 2.3, 32.1 ± 1.8, and 45.2 ± 4.1 s for 0.25-, 0.50-, and 0.75-G_z_, respectively. The mean G_z_-levels measured across the 3 flight days were 0.25 ± 0.02, 0.50 ± 0.02, and 0.75 ± 0.02 G_z_. The G_z_-level during straight-and-level flight preceding each parabola was 1.00 ± 0.05 G_z_ (Figure 2).

### Blood Pressure and Heart Rate

Mean arterial blood pressure and heart rate by G_z_-level are presented in Table 2. Mean arterial pressure during 1.00-G_z_ seated was significantly greater than mean arterial pressure during 0.75-G_z_, 0.50-G_z_, and 0.25-G_z_. Similarly, heart rate during 1.00-G_z_ seated rest was greater than heart rate during 0.50-G_z_ and 0.25-G_z_. We were unable to detect differences in heart rate when comparing 1.00-G_z_ seated to 0.75-G_z_.

**Table 2:** Mean heart rate and blood pressure while seated in 1.00-G_z_ and during partial gravity.

	Mean	95% CI	Difference from 1-G_z_	95% CI	*p*
**Heart rate (bpm)**			
1-G_z_ Seated	60	(51, 68)	–	–	–
0.75-G_z_	58	(51, 66)	−2	(−5, 1)	0.256
0.50-G_z_	56	(49, 63)	−4	(−7, −1)	0.015
0.25-G_z_	56	(49, 62)	−4	(−7, −1)	0.006
**Mean arterial pressure (mmHg)**			
1-G_z_ Seated	95	(79, 111)	–	–	–
0.75-G_z_	89	(74, 103)	−7	(−9, −4)	<0.001
0.50-G_z_	85	(66, 103)	−10	(−16, −5)	<0.001
0.25-G_z_	80	(66, 94)	−15	(−17, −13)	<0.001

### Vascular Responses

**Figure 3:**
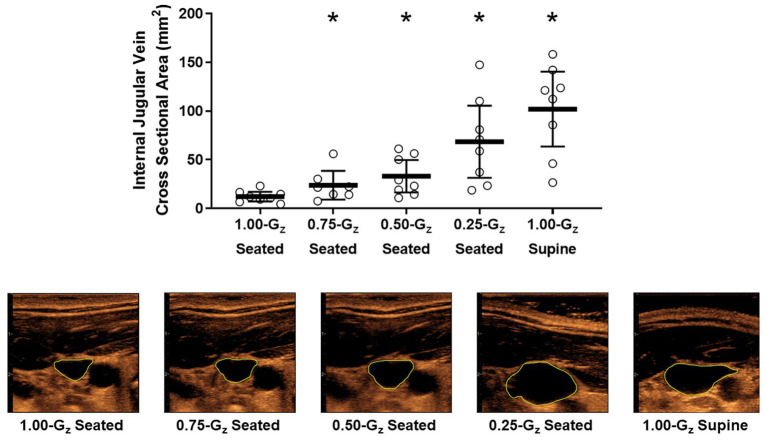
Internal jugular vein (IJV) cross-sectional area (**upper panel**) and representative images from 1 subject (**lower panel**) acquired by ultrasound during seated rest across different G_z_-levels and during 1.00-G supine rest. Open circles represent individual data. Black bar represents mean and 95% confidence interval. ^*^Significantly different than seated 1.00-G (*p* < 0.05).

Vascular dimensions and flow by G_z_-level are presented in Table 3. IJV cross-sectional area during 1.00-G_z_ seated was significantly smaller than at all evaluated partial gravity levels (Figure 3). In contrast, we were unable to detect differences in IVC diameter between measurements acquired during 1.00-G_z_ seated and any level of partial gravity or during 1.00-G_z_ supine. CCA blood flow during 1.00-G seated was significantly less than 0.75-G_z_ and 1.00-G_z_ supine, but differences were not detected at partial gravity levels 0.50-G_z_ and 0.25-G_z_. Differences in the average CCA cross-sectional area were not detected between any of the evaluated conditions.

**Table 3:** Mean vascular dimensions and flow while seated in 1.00-G_z_ and partial gravity and while supine in 1.00-G_z_ (0-G_z_ analog).

	Mean	95% CI	Difference from 1-G_z_	95% CI	*p*
**IJV area (mm^2^)**			
1-G_z_ Seated	12	(9, 16)	–	–	–
0.75-G_z_	24	(13, 35)	12	(1, 23)	0.032
0.50-G_z_	34	(21, 46)	21	(8, 35)	0.002
0.25-G_z_	68	(40, 97)	56	(27, 85)	<0.001
1-G_z_ Supine	108	(74, 131)	91	(64, 118)	<0.001
**IVC diameter (cm)**			
1-G_z_ Seated	1.68	(1.45, 1.92)	–	–	–
0.75-G_z_	1.66	(1.44, 1.88)	−0.02	(−0.20, 0.15)	0.783
0.50-G_z_	1.81	(1.61, 2.02)	0.13	(−0.01, 0.27)	0.071
0.25-G_z_	1.69	(1.46, 1.91)	0.01	(−0.02, 0.22)	0.955
1-G_z_ Supine	1.81	(1.57, 2.05)	0.12	(−0.10, 0.35)	0.280
**CCA flow (ml/min)**			
1-G_z_ Seated	786	(635, 837)	–	–	–
0.75-G_z_	895	(686, 1,103)	159	(24, 294)	0.021
0.50-G_z_	845	(650, 1,039)	109	(−82, 300)	0.265
0.25-G_z_	826	(688, 963)	90	(−4, 184)	0.060
1-G_z_ Supine	836	(783, 891)	101	(21, 181)	0.013
**CCA cross-sectional area (cm)**			
1-G_z_ Seated	0.33	(0.31, 0.35)	–	–	–
0.75-G_z_	0.33	(0.31, 0.35)	0.01	(−0.01, 0.02)	0.350
0.50-G_z_	0.33	(0.30, 0.36)	0.00	(−0.01, 0.01)	0.466
0.25-G_z_	0.34	(0.31, 0.36)	0.01	(0.00, 0.02)	0.118
1-G_z_ Supine	0.32	(0.29, 0.34)	−0.01	(−0.02, 0.00)	0.147

**Figure 4:**
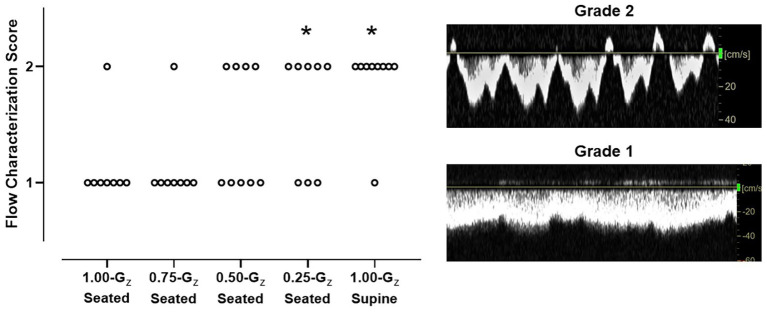
IJV flow characterization scores during 1.00-G_z_ supine rest (0-G surrogate) and during seated rest across G_z_-levels. Scores for each individual subject are represented. IJV flow was categorized by two expert sonographers using a 4-point scale, from continuous forward flow to stasis (Marshall-Goebel et al., 2019), but IJV flow in these subjects scored only to be either 1 (continuous forward flow) or 2 (pulsatile forward flow). Scoring differed between the two sonographers for only one subject at 1 G-level. Scores only from one rater (senior level sonographer) are shown. *n* = 9 for 0.50-G_z_ and 1.00-G_z_ supine; and *n* = 8 for 1.00-G_z_ seated, 0.75-G_z_ seated, and 0.25-G_z_ seated. ^*^Distribution of IJV flow scores significantly different (*p* < 0.05) than 1.00-G_z_ seated.

During 1.00-G_z_ seated and 0.75-G_z_ seated, the IJV waveform was characterized as continuous forward flow (score = 1) in all but one subject in each condition (Figure 4). At lower G_z_-levels, pulsatile forward flow (score = 2) became more apparent. Waveforms in four of nine, five of eight, and seven of eight subjects were scored as two at 0.50-G_z_ seated, 0.25-G_z_ seated, and 1.00-G_z_ supine, respectively. IJV flow scores were not different than 1.00-G_z_ seated at 0.75-G_z_ [*OR* = 1.0 (95% CI: 0–24), *p* = 1.00] and at 0.50-G_z_ [*OR* = 5.6 (95% CI: 1–38), *p* = 0.08], but IJV flow scores were significantly different than 1.00-G_z_ seated when compared to 0.25-G_z_ [*OR* = 12 (95% CI: 1–102), *p* = 0.026] and 1.00-G_z_ supine [*OR* = 56 (95% CI: 4–862), *p* = 0.004]. Waveforms corresponding to grades 3 and 4 were not observed in our subjects during parabolic flight.

**Figure 5:**
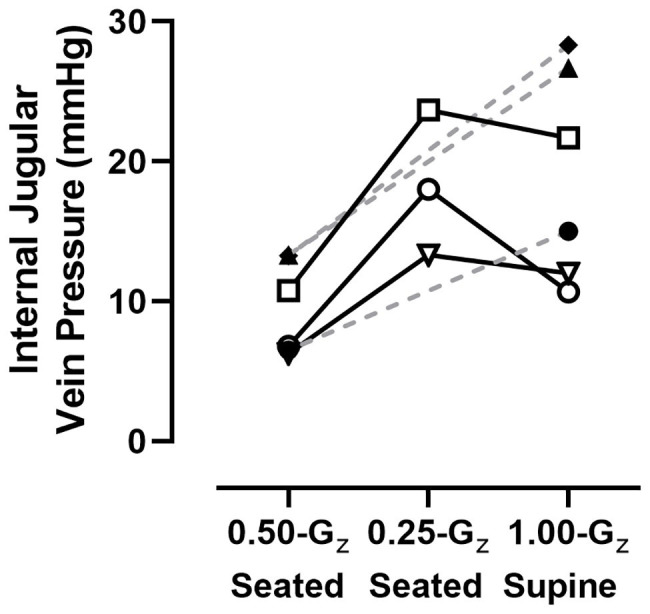
IJV pressure measured in six subjects using compression sonography. Individual results represented here are the mean of at least three measurements for each condition. Dashed lines are provided for subjects with data only from 1.00-G_z_ supine and 0.50-G_z_ seated to illustrate trends within those individual subjects and not meant to suggest predicted values at 0.25-G_z_.

One of the investigators (DSM) visually inspected all IJV pressure measurements to ensure the technical quality of the imaging before analyses were performed. This approach determined that data collected while subjects were seated at 0.75‐ and 1.00-G_z_ were not technically adequate, but data were available for six subjects during three conditions: preflight supine in 1.00-G_z_ and while seated at 0.25‐ and 0.50-G_z_. Individual data represented in Figure 5 are the mean of at least three IJV pressure measurements. In the three subjects with technically-adequate images in all three conditions, there appeared to be minimal difference in IJV pressure from 1.00-G_z_ supine to 0.25-G_z_ seated for two subjects and an increased IJV pressure in one subject. However, IJV pressure at 0.50-G_z_ seated (9.5 ± 3.4 mmHg) was lower than 1.00-G_z_ supine (19.1 ± 7.6 mmHg) for all five subjects [difference: 9.6 (95% CI: 5.0–14.1), *p* = 0.003].

## Discussion

The principal finding from this study was that compared to measurements acquired in the seated posture in 1.00-G_z_, IJV cross-sectional area increases and IJV flow pattern becomes pulsatile as the G-level decreases during brief periods of partial gravity produced by parabolic flight. There are two important perspectives that can be derived from these results. First, our data suggest that the minimum G_z_-level required to preserve hydrostatic conditions in the venous system of the upper body close to the upright 1.00-G_z_ posture, preventing IJV engorgement and changes in IJV flow patterns associated with the headward fluid shift in weightlessness, is greater than 0.50-G_z_. Second, our data suggest that G_z_-levels on the Moon (0.16-G_z_) and Mars (0.38-G_z_) would not be sufficient to prevent the headward distribution of venous blood that distends and changes the basic flow characteristics of the IJV. Future work will be required to verify that these observations during acute partial gravity are representative of the effects of chronic exposures (Shelhamer, 2016) and to determine whether partial gravity exposures during long-duration stays on the Moon and Mars will be protective against the risks of SANS and IJV thrombosis relative to weightlessness.

### Systemic Hemodynamics

We report here for the first time the heart rate and blood pressure responses across the range from 1.00‐ to 0.25-G_z_, although measures have been obtained during parabolic flight simulating lunar and Martian gravity (Widjaja et al., 2015; Beck et al., 2018). Lower mean arterial pressure has been reported previously while seated in 0-G produced by parabolic flight, likely resulting from increase in central blood volume (Lathers et al., 1989; Mukai et al., 1991), reduced sympathetic activity, and peripheral vasodilation (Iwase et al., 1999; Ogoh et al., 2015), but no similar data are available across this range of partial gravity levels. In apparent agreement with studies in weightlessness, mean arterial pressure appeared to decrease as G_z_-level decreased in this study presumably as the headward in fluid shift increased. We also observed that heart rate was lower during the two lowest partial gravity levels. Reduced heart rate during 0-G has not been consistently observed in previous parabolic studies (Lathers et al., 1989; Mukai et al., 1991), perhaps, because of differences in body postures or the duration of parabolas. In general, these responses are consistent with a centralization of blood volume.

### Arterial Flow to the Head

When considering the effects of partial gravity levels on cranial venous parameters, one must also consider the effects of cranial arterial flow. In this study, we did not observe an effect of G_z_-level on CCA diameter; although CCA flow was higher during 1-G_z_ supine than during 1-G_z_ seated, similar to previous observations (Sato et al., 2012; van Campen et al., 2018), the effects during partial gravity levels were not consistent. While an increase in CCA flow might be suggestive of elevated cerebral blood, middle cerebral artery flow velocity does not increase during weightlessness produced by parabolic flight due to systemic vasodilation (Ogoh et al., 2015), even though central blood volume and cardiac output may be higher (Ogoh et al., 2005). Although not examined in this study, we have no reason to suspect that the cerebral autoregulation was altered during these partial gravity levels.

### Venous Drainage From the Head

Previous observations during spaceflight (Arbeille et al., 2015; Marshall-Goebel et al., 2019) and weightlessness during parabolic flight (Martin et al., 2016) are consistent with venous congestion, and in this study, as the G_z_-level deceased during parabolic flight, a graded expansion of the IJV was observed. The blood volume located above the heart level (Lawley et al., 2017) likely increased as G_z_-level decreased due to a reduced hydrostatic pressure gradient between the head and the heart. Caudal fluid shifts during parabolic flight when transitioning from 1.00-G_z_ to 0-G_z_ are evident from measures of lower limb circumference (Bailliart et al., 1998) and thoracic impedance (Mukai et al., 1991), but no similar data have been acquired during these novel partial gravity conditions to explain our IJV cross-sectional area findings. However, manipulation of the hydrostatic gradient in a ground-based study using graded head-up tilt angles (Valdueza et al., 2000) produced a similar effect on IJV cross-sectional area as during this partial gravity parabolic flight experiment. In this study, mean IJV cross-sectional area increased almost 10-fold from 1.00-G_z_ seated to 1.00-G_z_ supine, but individual variation between subjects was clear, as is variation in the magnitude of the response between studies (Valdueza et al., 2000; Lawley et al., 2017; Marshall-Goebel et al., 2019). Location of the measurement along the IJV is one source of variation (Magnano et al., 2016; Marshall-Goebel et al., 2018), but images were obtained in similar sites. Individual variability in the response of the IJV cross-sectional area to posture changes likely reflect individual differences in anatomical structures, IJV valve patency, and vessel function, including between-subject differences in upper body venous compliance.

Though in this experiment we sought to describe the response of IJV cross-sectional area across the range of G_z_-levels, this parabolic flight campaign did not include 0-G parabolas. Reduced weighting of tissue overlying the IJV might contribute to IJV expansion during partial gravity by increasing transmural pressure compared to 1.00-G_z_ supine, similar to the explanation postulated for the decrease in central venous pressure during weightlessness (Buckey et al., 1993, 1996; Foldager et al., 1996; Videbaek and Norsk, 1997), and thus, using measures acquired during 1.00-G_z_ supine as our 0-G analog may have underestimated the effects. The IJV is a low pressure vessel with high compliance such that even small changes in pressure would result in large changes in IJV dimensions (Magnano et al., 2016). Others (Arbeille et al., 2015; Lawley et al., 2017) reported that the IJV is enlarged during weightlessness beyond that measured during supine rest in 1.00-G_z_. In contrast, we recently reported (Marshall-Goebel et al., 2019) that IJV cross-sectional area measurements during spaceflight were not different than those measured while supine before spaceflight.

While IJV cross-sectional area during full and partial gravity appears to represent a continuum across 1.00-G seated and partial G_z_ levels, the flow with the IJV may not follow this same pattern. We observed normal, continuous, or pulsatile forward IJV flow in all the subjects during seated and supine 1.00-G_z_, but during parabolic flight we observed a G_z_-level dependent transition from forward flow in the 1.00-G_z_ seated posture to pulsatile flow in partial gravity. Mean IJV cross-sectional area increased ~100% from 1.00-G_z_ seated to 0.75-G_z_ seated with no corresponding change in IJV flow character, and the transition from continuous forward to pulsatile flow did not occur in more than half of the subjects until 0.50-G_z_ seated when mean IJV cross-sectional area had almost tripled. The G_z_-level at which the transition occurred was not the same across subjects, but in general was consistent once the flow pattern became pulsatile as the G_z_-level decreased. Transition of the flow pattern likely results from filling of the vessel and stretching of the vein wall to an individual’s threshold such that the energy transmitted from cardiac contractions and respiration are more easily transmitted across the continuous fluid column (Anliker et al., 1969) and reflected in the character of IJV flow.

In the three gravity conditions in which technically sound measures of IJV pressure were acquired, it was apparent that IJV pressure decreases as G_z_-level increases. In the 1.00-G seated condition, the IJV is not engorged (Valdueza et al., 2000; Cirovic et al., 2003), as reflected in our IJVA measures, and thus, even small amounts of externally-applied pressure will compress the vessel making the measurement difficult. In our experience in the laboratory under more controlled conditions, IJV pressure measured with compression sonography when seated in normal gravity is between 0 and 10 mmHg (Marshall-Goebel et al., 2019). IJV pressure at 0.75-G_z_ appears to be similarly difficult but the measurement was easier to acquire when the vessel became engorged and easier to visualize at lower G-levels and while supine. Our measurements in 1.00-G_z_ supine are between that which we previously reported during supine in 1.00-G_z_ (9.9 ± 5.1 mmHg) and while seated in 0-G_z_ (23.9 ± 5.6 mmHg; Martin et al., 2016). The difference between our current results and those we previously published likely reflects the smaller amount of data available in these subjects in combination with the individual variability that we previously observed with this measurement technique (Martin et al., 2015). Because the methodology requires compression of the overlying tissues to compress the vein, between-subject differences could arise because of differences in tissue stiffness, thickness in subcutaneous tissues, or the location chosen along the IJV to conduct compression measures. However, given the consistency of the response between 1.00-G_z_ supine and 0.50-G_z_ seated, we are confident that the pattern of IJV pressures presented here reflects actual pressure changes within the IJV during partial gravity conditions. We observed a similar trend in non-invasive IJV pressure measurements during our previous parabolic flight campaign in two subjects (Martin et al., 2016); IJV pressure was highest during 0-G_z_, decreased somewhat at 0.16-G_z_ (lunar gravity), but decreased by ~50% at 0.38-G_z_ (Martian gravity).

Importantly, these data were collected during acute exposures, and thus, it is not clear what effects will be observed during chronic stays in weightlessness or partial gravity when IJV dimensions, pressures, and flow patterns would be chronically altered compared to the normal exposures in Earth gravity. For example, the IJV flow pattern was normal in 11 astronauts before spaceflight, transitioning from continuous forward flow when seated to pulsatile flow when supine, but the sustained headward fluid shift might promote progression to more abnormal flow patterns in some individuals. After ~50 and ~150 days of spaceflight, while most observations of IJV flow were pulsatile (11 of 21 observations), some were stagnant (7 of 21 observations) or reversed direction (2 of 21 observations; Marshall-Goebel et al., 2019). Given that venous stasis is a risk factor for thrombus formation (Previtali et al., 2011) and that an occlusive thrombus has been observed in one astronaut and suspected in a second (Marshall-Goebel et al., 2019), maintenance of normal IJV hemodynamics during sustained exposures to weightlessness or partial gravity are likely important to crew health and performance.

A potential confounding factor to consider when interpreting IJV cross-sectional area, flow characteristics, and pressure during parabolic flight is the potential change in the vessels carrying cranial venous outflow in different G_z_-levels (Valdueza et al., 2000; Zivadinov and Chung, 2013). For example, in an upright posture in 1.00-G_z_ when venous outflow is assisted by gravity, drainage of the head occurs primarily through the vertebral veins, and the IJV is largely collapsed; vertebral vein flow is three times greater than that in the IJV, and IJV cross-sectional area is 10–15% of that which was measured while supine (Valdueza et al., 2000; Cirovic et al., 2003). As subjects are tilted toward supine, vertebral venous flow progressively decreases while IJV cross-sectional area and flow increases, such that venous outflow occurs predominantly through the IJV. To our knowledge, no similar data exist with which to compare flows and diameters across different veins during weightlessness and partial gravity.

Previously we suggested that changes in the IJV hemodynamics might be influenced directly by venous return from the lower body (Martin et al., 2016); increased venous return from the lower body with little change or decreased flow through the superior vena cava could contribute to increased right atrial filling and IJV distension during parabolic flight. In this study, we measured IVC diameter which has been used clinically as an index of fluid status and venous return. IVC diameter is decreased in individuals with low blood volume (Mandelbaum and Ritz, 1996) and decreased further upon standing in subjects who suffer from orthostatic intolerance (Ishizaki et al., 2004). We observed that IVC diameter generally was unchanged across G-levels, and subjects did not report any symptoms of motions sickness or orthostatic hypotension during the parabolic maneuvers. While these results suggest that IJV hemodynamics is not directly influenced, measurement of flow from the IVC is required to confirm this.

### Countermeasures

Given that the weightlessness-induced headward fluid shift and associated IJV congestion are hypothesized to be contributing factors to the risk of SANS (Stenger et al., 2017) and IJV thrombosis (Marshall-Goebel et al., 2019), countermeasures that reverse the headward fluid shift may be protective of astronaut health during long-duration spaceflight. In addition to artificial gravity through short-arm centrifugation (Clément et al., 2015), countermeasures that have been proposed for the purpose of redistributing fluids to the lower body and relieving venous congestion during spaceflight include lower body negative pressure (Watkins et al., 2017; Marshall-Goebel et al., 2019), occlusive thigh cuffs (Balasubramanian et al., 2018), and exercise (Scott et al., 2019). An impedance threshold breathing device, which increases the resistance to inspiration and thus creates more negative pressure in the chest, also might assist with reducing IJV congestion (Convertino et al., 2005).

This study provides evidence that countermeasures targeting the prevention of the weightlessness-induced headward fluid shift will need to produce hemodynamic effects in excess of that created by 0.50-G_z_ to meaningfully reduce venous congestion. Compared to 1.00-G_z_ supine, our 0-G analog, exposure to 0.25-G_z_ produced a 30% reduction in IJV cross-sectional area and a mild reduction in IJV pressure; yet, the majority of subjects still exhibited a pulsatile IJV flow pattern. At 0.50-G_z_, IJV cross-sectional area was further reduced and IJV pressure approached that measured during 1.00-G_z_ seated, but still IJV flow was pulsatile in four of nine subjects. However, at 0.75-G_z_, IJV flow was continuous in seven of eight subjects, and IJV cross-sectional area was reduced from 0-G by 80%. It is important to note, however, that our data result from an acute exposure and that we did not observe any IJV waveforms representative of stagnant (grade 3) or reverse flow (grade 4) at lower G-levels. Thus, from these data, it is not possible to ascertain whether partial gravity exposures would restore normal, forward, or pulsatile IJV flow once stagnant or reverse flow patterns have developed during prolonged weightlessness. In fact, in only three of the seven cases, in which stagnant or reverse flow was observed after ~50 and ~150 days of spaceflight was lower body negative pressure at 25 mmHg of decompression, a level of lower body negative pressure does not approximate 1.00-G_z_ levels of orthostatic stress (Wolthuis et al., 1974), effective in restoring forward or pulsatile IJV flow (Marshall-Goebel et al., 2019). Thus, astronauts spending extended periods of time in lunar (0.16-G_z_) and Martian (0.38-G_z_) may not be at a reduced risk of SANS and IJV thrombosis when in these partial gravity environments. Recently, Baranov et al. (2016) reported, there was insufficient evidence to conclude that IJV diameter measured in six subjects after a simulated 3-week lunar mission [consisting 1 week of continuous 6° head-down tilt bed rest followed by 2 weeks of bed rest in which the subjects were horizontal (0° of tilt) for 8 h during sleep and at 9.6° of head-up tilt for 16 h during the day] differed from the IJV diameter of five subjects who underwent 3 weeks of simulated weightlessness (continuous 6° head-down tilt bed rest). Thus, countermeasures that would be employed by astronauts while weightless during transit to and from their destination also may be required during stays on these extraterrestrial surfaces.

### Limitations

Several limitations of this study should be acknowledged. First, IJV measures were acquired on the right side only, and we did not determine left or right side IJV dominance in these subjects. Thus, we cannot comment as to the hemodynamics on the left IJV, which generally has a smaller diameter than the right side and may have lower distensibility (Magnano et al., 2016) or the combined effects on venous flow from both vessels. Second, it is important to note that data acquisition during parabolic flight occurred while the subjects were in the upright, seated posture, so that the hydrostatic column in the G_z_ axis would be influenced by the different G_z_-levels in the same way that they would be during an artificial gravity countermeasure in weightlessness and during habitation of low gravity environments. This posture, however, has the potential to exaggerate fluid shifts during parabolic flight, in comparison to the fluid shifts associated with steady-state condition during longer partial gravity exposures (Norsk et al., 1987; Pantalos et al., 1999; Petersen et al., 2011). Abdominal organs and the volume of blood trapped in the abdomen when seated during hypergravity would be expected to move rapidly headward during the transition to reduced gravity, but these effects have not been measured in partial gravity conditions like those in this campaign. Third, we did not specifically control or measure fluid intake or hydration of our subjects, which may have contributed to the between-subject variability of the response to partial gravity. Hydration had a graded effect on right and left atrial pressures and volumes during weightlessness in instrumented non-human primates in parabolic flight (Latham et al., 1994), particularly right atrial pressure in the upright posture when the animals were volume depleted. Finally, these analyses do not control for the seated height of our test subjects, which could contribute to differences in hydrostatic pressure gradients across individuals, although in our statistical design subjects served as their own controls.

## Conclusions

We report for the first time that IJV cross-sectional area increases and the IJV flow pattern becomes more pulsatile as the G_z_-level decreases during partial gravity parabolic flight. While there is no clear answer yet as to the amount of caudal fluid shift required to mitigate the risk of SANS and IJV thrombosis during long-duration spaceflight, these results suggest that G_z_-levels greater than 0.50-G_z_ will be necessary to be protective and that there may be a risk of SANS and IJV thrombosis in the lunar and Mars environments. Validation of a countermeasure prescription (magnitude of fluid shift reversal, frequency, and duration) during long-duration spaceflight and assessment of the cumulative response to prolonged habitation in a partial gravity environment are required to substantiate these assertions. Inflight monitoring of the caudal fluid shifts and the effectiveness of countermeasures will be helpful in optimizing the countermeasure prescription and efficacy.

## Data Availability Statement

Requests to access the datasets should be directed to NASA’s Life Sciences Data Archive (https://lsda.jsc.nasa.gov/).

## Ethics Statement

The studies involving human participants were reviewed and approved by French National Comité de Protection des Personnes and the NASA Johnson Space Center Institutional Review Board. The participants provided their written informed consent to participate in this study. Written informed consent also was obtained from all participants for the publication of any potentially identifiable images.

## Author Contributions

SMCL contributed to the study design and implementation, interpretation of results, drafting and revision of the manuscript, and approval of final draft. DM contributed to the study design and implementation (including oversight of ultrasound procedures, data collection, and analyses), editing of the manuscript, and approval of final draft. CM contributed to the study implementation (including engineering support and data collection), editing of the manuscript, and approval of final draft. JS contributed to the study design, editing of the manuscript, and approval of final draft. SSL contributed to the interpretation of results, editing of the manuscript, and approval of final draft. BM contributed to the interpretation of results, editing of the manuscript, approval of final draft, and secured funding. NM contributed to the statistical analyses, interpretation of the results, drafting and revision of the manuscript, and approval of final draft. LP-S and MS contributed to the study design, editing of the manuscript, approval of final draft, and secured funding. All authors contributed to the article and approved the submitted version.

### Conflict of Interest

SMCL, DM, CM, SSL, BM, and NM were employed by KBR, under the Human Health and Performance Contract to NASA, during the performance of this study.The remaining authors declare that the research was conducted in the absence of any commercial or financial relationships that could be construed as a potential conflict of interest.
